# Seroprevalence and associated risk factors for *Neospora caninum* infection in dairy cattle in South Africa

**DOI:** 10.1007/s00436-024-08309-8

**Published:** 2024-08-14

**Authors:** Whatmore Munetsi Tagwireyi, Peter N. Thompson, Gema Alvarez Garcia, Darshana Morar-Leather, Luis Neves

**Affiliations:** 1 Department of Production Animal Studies, Faculty of Veterinary Science, University of Pretoria, Pretoria, South Africa; 2 Clinical Sciences Department, Ross University School of Veterinary Medicine, Basseterre, St Kitts and Nevis West Indies; 3 SALUVET Group, Department of Animal Health, Veterinary Faculty, Complutense University of Madrid, Madrid, Spain; 4 Department of Veterinary Tropical Diseases, Faculty of Veterinary Science, University of Pretoria, Pretoria, South Africa; 5 Centro de Biotecnologia, Eduardo Mondlane University, Maputo, Mozambique

**Keywords:** *Neospora caninum*, Dairy cattle, South Africa, Risk factors, Seroprevalence, ELISA, Western blot

## Abstract

Bovine neosporosis is a widespread parasitic disease associated with significant economic losses. Its effects on the reproductive performance of cows have resulted in losses that run into the hundreds of millions of US dollars in dairy industries in various countries (Reichel et al., Int J Parasitol 43:133–142, 2013). Due to outdated and scant information on the occurrence of *Neospora caninum* infection in South Africa, the study aimed to determine the seroprevalence and risk factors associated with infection in dairy cattle in South Africa. A total of 1401 blood samples were randomly collected from cattle on 48 dairy farms in seven of the nine provinces in South Africa. A close-ended questionnaire was used in a cross-sectional study to obtain farm-level and animal-level data. Serological testing was done using a commercial IDvet Screen® *Neospora caninum* Indirect ELISA. An overall seroprevalence, adjusted for test sensitivity and specificity, of 2.3% (95% CI, 1.3–4.1) was detected and 48% (23/48) of sampled farms had at least one animal testing positive. The highest seroprevalence of *N. caninum* was in the KwaZulu-Natal province with 7.5% (95% CI, 3.8–14.3), and the lowest in Western Cape with 0.1% (95% CI, 0–1.2). The highest within-farm seroprevalence of 25% was detected on a farm in the North West Province. In a multivariable logistic regression model, the odds of *N. caninum* seropositivity were higher in Holstein–Friesian cattle when compared to other breeds. Good hygiene was identified as a protective factor. Cattle left out on pasture had increased odds of testing positive for *N. caninum* compared to those that were penned. The odds of testing seropositive for *N. caninum* was higher on farms that practised segregation of cattle into different age groups. The purchase of replacement animals was a significant risk factor, as open herds had increased odds of *N. caninum* seropositivity. Cattle on farms that did not have a specific calving location were more likely to be seropositive. This is the first such study in South Africa and shows that *N*. *caninum* is widely distributed in the country at a low seroprevalence, but it may be a cause of concern on certain farms.

**Supplementary Information:**

The online version contains supplementary material available at 10.1007/s00436-024-08309-8.

## Introduction

*Neospora caninum* is a globally distributed apicomplexan protozoan that has become one of the more important aetiologies of parasitic abortion in cattle and causes clinical disease in canids (Anderson et al. 2000; Lindsay and Dubey 2000). Numerous studies have approximated the annual losses due to neosporosis for the dairy industries in North America to be around US $842.9 million, in South America around US $194.4 million, US $54 million in Australia and US $32.4 million in the UK (Reichel et al. 2013). These losses are further compounded by the fact that bovine neosporosis control interventions revolve around serodiagnosis and herd management as there is no economically viable and efficacious treatment or vaccine (Reichel et al. 2014; Dubey et al. 2017).

Vertical transmission from infected dam to offspring is the most important route of *N. caninum* infection in cattle, with horizontal transmission via ingestion of sporulated oocysts also playing a crucial role (Llano et al. 2018; McAllister 2016). During vertical transmission, endogenous transplacental transmission through reactivation of quiescent bradyzoites during pregnancy has been shown to predominate over exogenous transplacental transmission that occurs after primoinfection during pregnancy (Williams et al. 2009). Domestic and wild canids are definitive hosts for the parasite and are considered essential in transmission and maintenance of infection in dairy herds (Lindsay and Dubey 2000; Dubey and Schares 2011; Dubey et al. 2017). They facilitate horizontal transmission by contaminating feed and water sources with oocysts, which become infective once they have sporulated. Three significant abortion patterns of the infection have been documented in bovines: sporadic, endemic (increased abortions) and epidemic (outbreaks), with the latter being more economically devastating to farmers in the short to medium term (Wouda et al. 2000; Dubey et al. 2017). Diagnosis of *N. caninum* infection in cattle is routinely done by means of various serological tests to detect the presence of specific antibodies against the parasite, which are indicative of exposure, and suggests that infection is unlikely to clear spontaneously (Björkman and Uggla 1999). Numerous serological tests have been developed but enzyme-linked immunosorbent assays (ELISAs) are frequently commercialized and well validated (Alvarez-García et al. 2013).

The seroprevalence of *N. caninum* in cattle in Africa has been reported to range from 1.6 to 35.1% (VanLeeuwen et al. 2021). Low seroprevalences of 1.6–5.7% have been reported in cattle in South Africa, Nigeria and Namibia (Adesiyun et al. 2020; Ayinmode et al. 2017; Samkange et al. 2023). Moderate seroprevalences have previously been reported in some African countries including South Africa (9%), Ethiopia (13.3%), Senegal (17.9%) and Algeria (19.6%) (Njiro et al. 2011; Ghalmi et al. 2012; Asmare et al. 2013; Kamga-Waladjo et al. 2010). Extremely high seroprevalences of 25.6% and 35.9% were reported in Kenya and Egypt, respectively (Gaber et al. 2021; Okumu et al. 2019).

South Africa has approximately 891 dairy farms, and 85.8% of the country’s milk production originates predominately from the coastal regions, namely Western Cape (29.3%), Eastern Cape (28.5%) and KwaZulu-Natal (28%) (Milk Producers Organisation 2023). Dairy farming in South Africa is under substantial economic pressure, and forecasts indicate that these challenges may worsen. It is therefore important to investigate issues that adversely affect productivity. Productivity and reproductive parameters such as duration of service, age at first calving and number of services or inseminations per conception have been shown to rise with *N. caninum* seropositivity on dairy farms (de Barros et al. 2021). There is a lack of information regarding *N. caninum* infection in dairy cattle in South Africa. However, two studies have been conducted in beef cattle. One study in Gauteng reported a seroprevalence of 9% in 239 cattle (Njiro et al. 2011), while another study in 184 communally grazed cattle in Mpumalanga province near the Kruger National Park reported a seroprevalence of 1.6% (Adesiyun et al. 2020). A clinical case was also reported in a white rhinoceros calf in a game-breeding centre in the North West Province (Williams et al. 2002). This underscores the parasite’s potential impact on wildlife and the epidemiological role of wildlife in the transmission of infection at the wildlife-livestock-human interface. This study aimed to determine the seroprevalence and associated risk factors of *N. caninum* in dairy cattle from seven of the nine provinces in South Africa.

## Materials and methods

### Location

The study was carried out in seven of the nine provinces of South Africa (Western Cape, Eastern Cape, Free State, Mpumalanga, Northwest, KwaZulu-Natal and Gauteng). The country stretches from 22°S to 35°S and from 17°E to 33°E and has a generally temperate climate with average temperature ranges of 15 to 36 °C in summer and − 2 to 26 °C in winter and an annual average rainfall of 464 mm (World Bank Group 2021). The major dairy farming areas are in the Western and Eastern Cape and KwaZulu-Natal, and the dairy farming system is characterized by either pasture-based or total mixed ration systems.

### Study design and sampling of cattle

Multi-stage sampling was used for the study, with the farm as the primary sampling unit, stratified by province, followed by random sampling of cattle within herds. The sample size to estimate a proportion with 95% confidence was calculated as follows (Thrusfield 2018):

$$n=({1.96}^{2}\times {P}_{exp}(1-{P}_{exp}))/{d}^{2}$$

where $n$ is the required sample size, ${P}_{exp}$ is the expected prevalence and $d$ is the desired absolute precision. Sample size was multiplied by the design effect (*D*) for multi-stage sampling, calculated as follows (Bennett et al. 1991):

$$D=1+\rho (m-1)$$

where $\rho$ is the intra-cluster correlation coefficient (ICC) and $m$ is the average cluster size.

Due to a lack of recent data on seroprevalence of *N. caninum* in dairy cattle in South Africa, an expected prevalence of 12% was used, based on previously reported seroprevalence in Africa and elsewhere (Dubey et al. 2007; Semango et al. 2019; Selim et al. 2023; Samkange et al. 2023). A desired precision of 5% was used. The ICC for *N. caninum* is unknown but for most diseases is unlikely to exceed 0.25 (Otte and Gumm 1997); therefore, using $\rho$ = 0.25 and $m$ = 30, $D$ was calculated as 8.25. Hence, the minimum required sample size was 1345 animals to be sampled from 45 farms.

Forty-eight farms were randomly selected from lists provided by veterinarians and farmers’ organisations working with dairy farmers in the study areas, with the number of farms selected in each province approximately proportional to the total number of dairy farms in that province. Up to 45 animals per farm, depending on the size of the farm, were selected using convenience sampling in order not to interfere with routine procedures on the farm while attempting to obtain a representative sample from the herd. Animals older than 6 months of age were sampled to avoid false positives due to the presence of colostral antibodies. Individual blood samples (5–10 ml) were collected using the coccygeal vein into serum tubes. The samples were transported at 4 °C; they were separated by centrifugation, and serum was stored at − 20 °C until analysis.

### Serological testing

All the analyses were performed at the University of Pretoria, Department of Veterinary Tropical Diseases, Research & Training Laboratories. *Neospora caninum* antibodies were detected by a commercial IDvet Screen® *Neospora caninum* Indirect ELISA performed according to the manufacturer’s instructions (IDvet 2021). The test uses an anti-ruminant conjugate; a sample to positive control ratio (S/P) percentage ≥ 50% is considered positive, 40–50% is doubtful and < 40% is negative (IDvet 2021). Additionally, the ELISA results were confirmed using Western blot (WB), and a sample was only considered positive with the observation of a clear 17–19 kDa antigenic fraction (Bartels et al. 2006; Álvarez-García et al. 2003). The WB was used as a confirmatory test to assess the presence or absence of antibodies against *N. caninum* and address the low specificity of the ELISA (Álvarez García et al. 2006). A herd was considered positive if at least one animal in the herd was seropositive (Bartels et al. 2006; Alvarez-Garcıa et al. 2002).

### Questionnaire survey

Interviews were conducted with owners and managers of each of the farms where sample collection took place. A semi-structured closed-ended questionnaire (Supplementary material 1 and 2) was completed at the time of sample collection to obtain information about potential risk factors. The questionnaire was developed after a thorough review of relevant literature on risk factors and in consultation with experts in the field (Dubey et al. 2007, 2017). Questions focused mainly on (1) the general farm characteristics, (2) host factors, (3) herd management and biosecurity factors, (4) reproduction health management and production parameters and (5) role of potential reservoir and intermediate hosts.

### Data analyses

All the analyses were performed in Stata® 17 (StataCorp, College Station, TX, USA.). Serological test results were interpreted in series, and a sample was considered positive if it was positive or doubtful on ELISA and positive on WB. Sampling fraction for each herd was calculated as the proportion of the herd sampled, and sampling weight was calculated as the inverse of the sampling fraction. Seroprevalences were calculated weighting each observation by the sampling weight to remove the potential bias due to unequal sampling fractions. Standard errors of the seroprevalence estimates were adjusted by using a robust variance estimator to account for the clustered sampling design, to produce 95% confidence intervals. These analyses were done using the “svy” commands in Stata 17. The diagnostic sensitivity of ELISA (Se_ELISA_) was assumed to be 0.996, and the diagnostic specificity (Sp_ELISA_) was assumed to be 0.989 (Alvarez-García et al. 2013). The diagnostic sensitivity of WB (Se_WB_) was assumed to be 0.8, and the diagnostic specificity (Sp_WB_) was assumed to be 1.0 (García‐Lunar et al. 2013). Sensitivity of the tests in series was calculated as Se = Se_ELISA_ × Se_WB_ = 0.797, and specificity was calculated as Sp = 1 − [(1 − Sp_ELISA_) × (1 − Sp_WB_)] = 1.0 (Thrusfield 2018). Seroprevalence estimates and their confidence limits were then adjusted for imperfect Se and Sp using the Rogan and Gladen adjustment (Rogan and Gladen 1978):

$$\text{TP}=\frac{\text{AP}+\text{Sp}-1}{\text{Se}+\text{Sp}-1}$$

where $\text{TP}$ is the true prevalence, $\text{AP}$ is the apparent prevalence, $\text{Se}$ is the test sensitivity and $\text{Sp}$ is the test specificity.

Univariate associations of potential risk factors with the outcome (*N. caninum* seropositivity) were assessed using a two-tailed Fisher’s exact test. These included factors plausibly on the causal pathway as well as potential confounders, but excluded factors considered to be potential consequences of *N. caninum* infection, such as abortion. Independent variables associated with the outcome with *p* < 0.2 were selected for inclusion in a multivariable logistic regression model. Predictors were sequentially dropped from the model beginning with the least significant, based on Wald’s *p*-value, until all remaining predictors were significant (*p* < 0.05). All predictors, including those not originally added and those already eliminated, were then each individually added to the model and retained if significant (*p* < 0.05). The fit of the final multivariable model was assessed using the Hosmer–Lemeshow goodness-of-fit test. Adjustment for clustering by herd and for sampling weights was done using the “svy” commands in Stata. For the presentation of the model, the base level for *province* was Western Cape; for *breed*, it was Jersey; and for ordinal (0/1/2/…) and binary (0/1) variables, it was 0.

## Results

### Farm characteristics

**Fig. 1:**
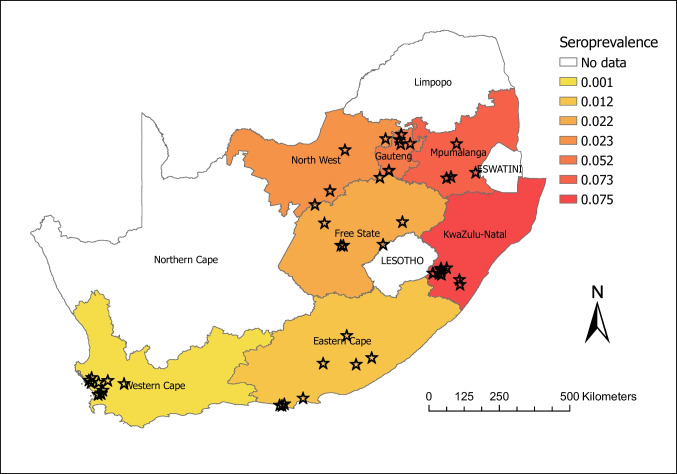
Map showing sampling locations and seroprevalence by province of *Neospora caninum* in dairy cattle in South Africa

A total of 1401 animals on 48 farms across seven provinces were sampled between February 2022 and September 2023 (Fig. 1). The average number of animals on the 48 farms was 1255 animals, ranging from 12 to 11,000 animals. Holstein–Friesian accounted for 34.9% (489/1401) of the animals sampled; Jersey accounted for 10.6% (149/1401); Holstein-Jersey crosses accounted for 10.0% (140/1401), and mixed breeds accounted for 44.5% (623/1401). The average herd age ranged from 3 to 5 years. Most of the farms sampled (33/48; 69%) were on a pasture-based production system, while 31% (15/48) were on a total mixed ration production system. Of the sampled farms, 85% (41/48) reported having dogs on the farm. The two farms most heavily infected with *N. caninum*, one in KwaZulu-Natal (17.9%) and the other in the North West Province (25%), reported having numerous reproductive problems, including high abortion rates and increased matings or inseminations per conception.

### Seroprevalence of *Neospora caninum*

ELISA was used as a screening test. A total of 1401 cattle serum samples were tested, and 63 (4.5%), were positive for the presence of antibodies against *N. caninum*. All positive and doubtful samples were tested further with Western blot as a confirmatory test. The criterium for a positive result was a clear recognition of a 17–19 kDa antigenic fraction. Western blot analysis confirmed seroreactivity against *N. caninum* immunodominant antigens in 45/63 (71%) of the ELISA-positive samples and 4/11 (36%) of the ELISA-doubtful samples (Table 1).

**Table 1:** Seroprevalence of *Neospora caninum* infection in dairy cattle in the milk-producing provinces of South Africa

Province	Number of farms sampled	Number of positive farms (%)	Number of animals sampled	Number of positive animals	Adjusted animal-level seroprevalence % (95% CI)*	Within-farm seroprevalence range %
Eastern Cape	8	2 (25)	204	2	1.2 (3.3–8)	0–4.0
Free State	5	3 (60)	166	4	2.2 (0.7–6.9)	0–5.7
Gauteng	6	2 (50)	180	3	5.2 (3.3–8)	0–5.0
KwaZulu-Natal	10	8 (80)	274	16	7.5 (3.8–14.3)	0–17.9
Mpumalanga	4	3 (75)	140	7	7.3 (4.3–12.1)	0–8.6
North West	5	4 (80)	187	16	2.3 (0.3–15.5)	0–25.0
Western Cape	10	1 (10)	250	1	0.1 (0–1.2)	0–4.0
**Total**	48	23 (48)	1 401	49	2.3 (1.3–4.1)	0–25.0

*Adjusted for sampling weight, clustering and test sensitivity and specificity

After adjusting for sampling weights and for test sensitivity and specificity, the true overall seroprevalence was estimated at 2.3% (95% CI, 1.3–4.1). Seroprevalence varied significantly between provinces (Fisher’s exact *p* < 0.001). All provinces had at least one positive farm, with 48% (23/48) of the farms having at least one seropositive animal. The lowest seroprevalence (0%) was reported on numerous farms across different provinces while the highest seroprevalence (25%) was reported on a farm in the North West Province (Table 1). Only one animal in the Western Cape tested positive.

### Potential consequences of *N. caninum* infection

Although animals in herds in which greater numbers of abortions had occurred showed somewhat higher *N. caninum* seroprevalence, this was not significant (*p* = 0.355). However, a positive association was found between the number of matings or inseminations per conception and the prevalence of *N. caninum* antibodies (*p* < 0.001), and there was also a positive association between a history of the birth of weak calves in the herd and *N. caninum* seropositivity (*p* = 0.046) (Table 2).

**Table 2:** Summary of *Neospora caninum* seroprevalence in dairy cattle in South Africa by categories of animal, farm and risk factor variables

Independent variable and level	Number of animals sampled	Number of positives	Seroprevalence (%)
Size of farm*			
< 10 hectares	87	10	11.5
10–100 hectares	163	6	3.7
101–500 hectares	503	17	3.4
> 500 hectares	648	16	2.5
Age of cattle*			
< 2 years	49	0	0
2–5 years	551	21	3.8
5–8 years	475	11	2.3
> 8 years	326	17	5.2
Breed of cattle*			
Holstein-Friesland	489	21	4.3
Jersey	149	1	0.7
Holstein-Jersey crosses	140	2	1.4
Mixed	623	25	4.0
Number of cattle on farm*			
1–100	111	10	9.0
101–300	223	9	4.0
301–1000	350	15	4.3
> 1000	717	15	2.0
Proximity to urban areas*			
< 5 km	142	10	7.0
5–10 km	348	13	3.7
> 10 km	911	26	2.9
Total mixed ration*			
No	939	41	4.4
Yes	462	8	1.7
Use of troughs*			
No	120	1	0.8
Yes	1281	48	3.8
Pasture drainage*			
Poor	221	5	2.3
Good	1045	43	4.1
Excellent	135	1	0.7
Level of hygiene*			
Poor	45	4	8.9
Good	976	36	3.7
Excellent	380	9	2.4
Presence of dogs*			
No	125	1	0.8
Yes	1276	48	3.8
Quarantine period*			
< 2 weeks	55	6	10.9
2–4 weeks	157	5	3.2
> 4 weeks	67	2	3.0
None	1122	36	3.2
Wild canids present*			
No	625	15	2.4
Yes	776	34	4.4
Rodent control*			
No	75	12	16.0
Yes	1326	37	2.8
Number of dogs on farm*			
< 2	160	1	0.6
2–5	100	0	0
5–8	568	24	4.2
> 8	573	24	4.2
Dogs used as cattle working dogs*			
No	1070	43	4.0
Yes	331	6	1.8
Calving location*			
No	215	14	6.5
Yes	1186	35	3.0
Calving location used as hospital*			
No	1154	46	4.0
Yes	247	3	1.2
Management of colostrum*			
Pool colostrum	541	24	4.4
Colostrum from dam	471	18	3.8
Both	389	7	1.8
Management of animal records*			
Software	1129	34	3.0
Log books	181	4	2.2
No recording	91	11	12.1
Birth of weak calves*			
No	471	10	2.0
Yes	930	39	4.2
Range of matings per conception*			
1–3	357	7	2.0
1–6	793	23	2.9
1–9	148	8	5.4
No records	91	11	12.1

*p < 0.2 and variable considered for the initial multivariable model

### Factors associated with *Neospora caninum* seropositivity

Twenty-three variables with *p* < 0.2 in the univariate analysis (Table 2) were initially included in the multivariable analysis. The final multivariable logistic regression model (Table 3) identified several factors significantly associated with odds of *N. caninum* seropositivity (*p* < 0.05). Holstein-Friesland cattle were 24.2 (95% CI, 2.7–220.8) times more likely than Jerseys to be *N. caninum* seropositive; mixed breed cattle were three times more likely to be seropositive, and Jersey cattle were the least likely to be seropositive. Cattle that were not penned were more likely to test positive to *N. caninum* when compared to cattle that were kraaled. Farms with poor hygiene in the milking parlour were more likely to be *N. caninum* positive compared to farms with excellent hygiene. Farms that practised segregation of cattle were more likely to be seropositive compared to farms that did not practise segregation of cattle. Open herd farms had higher odds of testing positive to *N. caninum* infection when compared to closed herd farms. Dairy farms that had a specific calving location were least likely to test positive for the parasite when compared to farms that did not use a specific calving location. The Hosmer–Lemeshow goodness-of-fit test indicated adequate model fit (*p* = 0.83).

**Table 3:** Final multivariable logistic regression model of factors associated with *Neospora caninum* seropositivity in dairy cattle in South Africa

Variable and level	Odds ratio (OR)	95% CI OR	*p*-value
Province			
Western Cape	1*	–	–
Gauteng	8.7	0.8–97.5	0.078
Eastern Cape	0.1	0.01–1.0	0.050
Northwest	2.6	0.1–55.3	0.528
Free State	10.2	1.03–101.1	0.048
Mpumalanga	47.6	3.9–577.7	0.003
KwaZulu-Natal	3.5	0.5–26.1	0.282
Breed of cattle			
Holstein–Friesian	24.2	2.7–220.8	0.006
Jersey	1*	–	–
Guernsey	0.1	0.02–1.2	0.071
Mixed	3.0	1.4–6.5	0.006
Cattle penned			
No	1*	–	–
Yes	0.1	0.01–0.1	< 0.001
Hygiene			
Poor	1*	–	–
Good	0.01	0–0.2	0.003
Excellent	0	0–0.07	0.001
Segregation of cattle			
No	1*	–	–
Yes	14.1	1.1–182.8	0.043
Closed herd			
No	1*	–	–
Yes	0.5	0.3–0.9	0.017
Calving location			
No	1*	–	–
Yes	0.1	0.03–0.8	0.024

Model equation: ln(p/(1 − p)) = 2.612 + 2.163 * province_GP_ − 2.432 * province_EC_ + 0.962 * province_NW_ + 2.321 * province_FS_ + 3.863 * province_MP_ + 1.255 * province_KZN_ + 3.187 * breed_H-F_ − 1.967 * breed_Guernsey_ + 1.101 * breed_mixed_ − 4.359 * penned − 4.763 * hygiene_good_ − 5.667 * hygiene_excellent_ + 2.647 * segregation − 0.689 * closedherd − 1.954 * calvinglocation, where p is the probability of being seropositive

*Reference level

## Discussion

The study found that *N. caninum* infection is widespread in dairy cattle in South Africa, although generally at a low seroprevalence. This is the widest serological study carried out in South Africa and revealed an overall seroprevalence of 2.3%, which is lower than reported in most studies in Africa. The two other studies in cattle in South Africa reported seroprevalences of 1.6% and 9% (Adesiyun et al. 2020; Njiro et al. 2011). Differences in seroprevalence within the country can be attributed to differences in animal management practises, differences in location and differences in type of animal production (beef vs dairy). Comparably low seroprevalences of 1.6–5.7% have been reported in cattle in South Africa, Nigeria and Namibia (Adesiyun et al. 2020; Ayinmode et al. 2017; Samkange et al. 2023). The parasite was observed to be widely distributed throughout South Africa with all the sampled provinces having at least one farm testing positive, but with substantial differences in seroprevalence between herds within the same province, ranging from 0 to 25%. This is consistent with most studies which show a wide geographical distribution of *N. caninum* infection in cattle across various localities within a country (Semango et al. 2019; VanLeeuwen et al. 2021; Bartels et al. 2006).

*Neospora caninum* seroprevalence was higher in the northern parts (North West Province) and eastern parts (KwaZulu-Natal Province) of the country. The eastern region of the country experiences warm and humid conditions, whereas the northern parts are drier but hotter, both creating suitable conditions for the sporulation of *N. caninum* oocysts. This is consistent with some studies which have shown that higher temperatures may facilitate faster sporulation of oocysts and are associated with higher *N. caninum* seroprevalence (Rinaldi et al. 2005; Dubey et al. 2007). The high *N. caninum* seroprevalence in the eastern parts of the country could also be associated with higher human population density. KwaZulu-Natal has the second highest human population density in the country (Department of Statistics South Africa 2023). A similar study in Germany showed that there is an increased risk of *N. caninum* seropositivity on farms located in areas with high human population density (Schares et al. 2003). It is thought that human population density is positively correlated to dog density, which in turn may increase the risk of *N. caninum* infection. Dogs play a significant epidemiological role in the spread of *N. caninum* infection on dairy farms (King et al. 2010; Dubey and Schares 2011). There are no official statistics on the dog population in South Africa. However, the high incidence of rabies outbreaks in dogs, livestock and humans along the eastern parts of the country could indicate the presence of substantial number of feral unvaccinated dogs and wildlife reservoirs of rabies particularly, including the black-backed jackal (Nel et al. 2009; Zulu et al. 2009). These could be another potential source of pasture contamination with *N. caninum* oocysts on farms in those areas. Though some wild canids have been confirmed as definitive hosts for the parasite, the role of African wild canids in the epidemiology of *N. caninum* should be investigated (Gondim et al. 2004; King et al. 2010; Dubey and Schares 2011).

Several biologically plausible factors were associated with the outcome in the univariate analysis but did not retain significance in the multivariable model. Proximity of farms to urban areas was associated with *N. caninum* seropositivity; this finding is complementary to the previous findings which use human density as an indicator for dog density (Dubey et al. 2007). This is also consistent with another study that identified proximity to town or a village as a significant risk factor for *N. caninum* infection (Hassig & Gottstein, 2002). Farms lacking rodent control measures reported higher *N. caninum* seroprevalence compared to those implementing rodent control. It is believed that parasite tissue cysts found in rodents might contribute to the sylvatic life cycle of *N. caninum* by serving as reservoir hosts for canids (Donahoe et al. 2015; Medina-Esparza et al. 2013). The presence of dogs on a farm was associated with *N. caninum* seropositivity, a well-documented risk factor for cattle infection (Paré et al. 1998; Wouda et al. 2000). As the number of dogs on farms increased, up to a maximum of eight, the risk of *N. caninum* infection also increased. This is likely because higher numbers of dogs on a farm increase the potential for environmental contamination with oocysts. A similar trend was observed in Egypt (Metwally et al.2023). Smaller farms showed a higher association with *N. caninum* seropositivity compared to larger farms. Similarly, farms with fewer animals also displayed higher *N. caninum* seropositivity compared to those with more animals. This observation may reflect differences in biosecurity and management practises, as smaller farms with fewer animals typically have fewer resources available to implement good farming practises.

Holstein–Friesian cattle had higher odds of testing positive to *N. caninum* infection compared to other breeds. Similar findings have been observed in other studies that may suggest that *Bos taurus* pure breeds are more susceptible to acquiring infection compared to crossbreeds (Fanta 2017; Asmare et al. 2013; Moore et al. 2009; Escalona et al. 2010; Bartels et al. 2006). Information on breed susceptibility to *N. caninum* infection is difficult to analyse due to potential confounding factors from farm management practises, which influence this association. Therefore, breed differences may be influenced by other unmeasured factors to some extent. Future research could explore genomic studies to investigate heritable traits related to susceptibility to neosporosis. The study revealed a significant correlation between the hygiene conditions of farms and the seropositivity to *N. caninum*, with seropositivity increasing as hygiene deteriorated. Poor hygiene, characterized by inadequate cleaning and disinfection practises in milking parlours and insufficient removal of slurry in pens, contributes to the contamination of water and feed with *N. caninum* oocysts. This finding aligns with previous studies that have identified good hygiene as a protective factor against *N. caninum* infection in cattle (Ghalmi et al. 2012; Abdeltif et al. 2022; Llano et al. 2018). Cattle that grazed on pasture were more likely to test positive to *N. caninum* compared to those kept in pens. This is likely because animals that spend most of their time on pasture have increased exposure to environments contaminated with oocyst. Studies in dairy cattle have shown the difficulty in preventing dogs, whether domestic or feral, from accessing pastures (Haddad et al. 2005). Other studies have also demonstrated an association between *N. caninum* seropositivity and grazing of cattle (Wei et al. 2022; Rinaldi et al. 2005). Future prospective studies could investigate the environmental contamination of feed, water and pastures with oocysts, as well as the potential interventions in dairy farming systems nationwide.

Cattle in closed herds had lower odds of testing positive to *N. caninum* compared to those in open herds, indicating that purchasing animals was a significant risk factor for *N. caninum* seropositivity. This could be attributed to the fact that newly acquired animals are not typically screened for *N. caninum* and may unknowingly introduce infection onto a farm. This finding is similar with other studies that have shown how introduction of new animals increases the risk of farm infection and the potential for vertical transmission of the parasite to future generations (Nasciutti et al. 2018; Gliga et al. 2022). The absence of a dedicated calving location on farms was significantly associated with *N. caninum* seropositivity, something that has been shown in numerous studies (Talafha and Al-Majali 2013). Farms lacking a designated calving area face higher risks of pasture contamination with blood, placenta, aborted foetuses and amniotic fluid, which are potential sources of infection. Without a dedicated calving location, proper disposal and disinfection of these materials become challenging and could increase the risk of horizontal transmission. This serves as an indicator of poor biosecurity practises on farms, leading to increased exposure of cattle to *N. caninum*. Interestingly, segregation of cattle into different age groups was associated with increased odds of *N. caninum* seropositivity. The reason for this association is unclear, and it may be due to other unmeasured factors related to animals in these groups having close contact and similar exposures.

The study revealed specific potential consequences linked to *N. caninum* infection associated with reproductive problems. It was observed that *N. caninum* was found to be prevalent in dairy farms experiencing increased matings or inseminations per conception, as well as a history of weak calf births. These consequences of *N. caninum* infection in cattle have been extensively documented in various studies (Dubey and Schares 2011; McAllister 2016).

In many studies, cost-effective in-house serological tests are commonly used for the diagnosis of *N. caninum*, despite their poor standardisation. Therefore, careful consideration should be given when interpreting the results from these tests. However, this study utilised a well-validated commercial ELISA with high sensitivity (99.6%) and specificity (98.9%), as evidenced by comparative evaluations with other commercially available ELISAs (Alvarez-García et al. 2013). Additionally, Western blot (WB) served as a confirmatory test to address specificity concerns associated with the ELISA. The study had several limitations, including incomplete coverage of the dairy cattle population in the country. Farms were selected from lists provided by veterinarians and farmers’ organisations, which resulted in under-representation of small-scale farms and those not associated with veterinarians or farmers’ organisations. It is likely that these may have been farms with poorer biosecurity measures and possibly higher *N. caninum* seroprevalence. Furthermore, *N. caninum*-positive animals do not always yield positive serology results, especially in cases of serial shedders, due to variations in host immunity (Anderson et al. 1997; Haddad et al. 2005), which was a significant constraint of this cross-sectional study. Enhanced diagnostics and improved access to them are essential for effective surveillance.

Despite a relatively low seroprevalence on dairy farms in South Africa, control of *N. caninum* should be integrated into regular herd health management programmes. The combination of low seroprevalence and variables associated with *N. caninum* infection related with biosecurity measures suggests that horizontal transmission could be relevant. A farm-specific, risk-based approach should be adopted, utilising the identified risk factors to implement effective herd health control programmes through education of farmers and veterinarians. It is crucial to implement control measures, in all infected farms particularly those with higher infection rates, as the absence of such measures can lead to an increase in transplacental transmission of infection over time, resulting in higher seroprevalence and abortion rates. Culling of infected cows that have confirmed *N. caninum* abortions within heavily infected herds should be considered. Farm biosecurity measures, including preventing access of dogs to the herds, feed and water, as well as prompt removal of aborted materials and after birth, are essential to reduce *N. caninum* transmission. Recommendations should also emphasize the monitoring of infection in replacement heifers prior to purchase and breeding to prevent transplacental transmission and persistence of chronic in utero infection. Active surveillance for *N. caninum* infection in bulk milk can also be instituted. In cases where the annual abortion rates are higher than 5%, it is recommended that the surveillance be made mandatory as this serological tool detects seropositive herds in lactating animals when individual prevalence is above 15–20% (Cirone et al. 2021; Enachescu et al. 2014). Enhanced diagnostic capabilities across the country would facilitate better implementation of screening and surveillance strategies. Future studies should evaluate the economic impact of *N. caninum* infection on affected farms in South Africa and investigate the main route of transplacental transmission in these herds to develop appropriate intervention strategies. Additionally, there is a necessity to explore *N. caninum* infection in beef cattle and wildlife to understand the epidemiological roles that wildlife play in the transmission of infection.

## Conclusions

*Neospora caninum* is widely distributed throughout the country, although with large variation between provinces. The large variation in farm-level seroprevalence of *N. caninum* suggests that various management risk factors could be responsible for this and need further investigation. Cows from herds that farmed with Holstein–Friesian breeds, penned cattle, had poor hygiene, practised cattle segregation according to age, had an open herd and did not use a dedicated calving location had a high likelihood of testing positive for *N. caninum* infection. Future studies could investigate various gaps in knowledge regarding *N. caninum* infection in South Africa, aiming to understand them better and implement appropriate interventions.

## Supplementary Information

Below is the link to the electronic supplementary material.

Supplementary file1 (PDF 188 KB)

Supplementary file2 (PDF 141 KB)

## Data Availability

No datasets were generated or analysed during the current study.
