# Supplementary material for: Seroprevalence and associated risk factors for Neospora caninum infection in dairy cattle in South Africa
Source: Parasitol Res. 2024 Aug 14;123(8):298. doi: 10.1007/s00436-024-08309-8 (PMC11324681; doi:10.1007/s00436-024-08309-8)
Supplement: Supplementary file 1 — Supplementary file1 (PDF 188 KB) [file 436_2024_8309_MOESM1_ESM.pdf]

UNIVERSITEIT VAN PRETORIA
UNIVERSITY OF PRETORIA
YUNIBESITHI YA PRETORIA

NEOSPORA CANINUM RISK FACTOR QUESTIONNAIRE

Date of sample collection: ____________________Study reference: ______________________________

Location (City, Municipality and Province):____

Address: ____

G.P.S coordinates: E______________________________ S____________________________________

Tel/Cell: ___________________________________ Email Address:_______________________________

1. Size of farm:

☐ < 10 hectares ☐ 10 – 100 hectares ☐ 101 – 500 hectares ☐ > 500 hectares

2. Total number of cattle on farm: ______________

☐ 1 – 100 (very small) ☐ 101 – 300(small) ☐ 301 – 1000(medium) ☐ > 1 000(large)

3. Proportion (% & numbers) of heifers, adult cattle and calves in the herd

☐ Heifers _________ ☐ Adult cattle _________ ☐ Calves ________

4. Average age of the herd

☐ < 3 years ☐ 3 – 5 years ☐ > 5 years

5. Breed of cattle:

☐ Holstein-Friesland ☐ Jersey ☐ Guernsey ☐ Ayrshire ☐ Dairy Swiss ☐ Mixed

6. Type production system:

☐ Commercial ☐ Non-commercial

7. Type of farm:

☐ Private ☐ Communal

8. Proximity to a town or village:

☐ Close (< 5kms) ☐ Far (5 - 10kms) ☐ Very far (>10kms)

Closest town or village is ________________________________ and ___________ kms away

9. Type of feeding regime:

☐ Pasture grazed ☐ Pasture grazed & zero grazing (TMR) ☐ Zero grazing (TMR)

10. Source of feed:

☐ Made on farm ☐ Made off farm / Commercial feed

11. Use of baled feed:

☐ Yes ☐ No

12. Use of troughs/ self-contained feeder for cattle feed and supplements:

☐ Yes ☐ No

13. Pasture drainage:

☐ Excellent (allows water to drain at a moderate rate without water pooling & puddling)

☐ Good (allows water to drain at a slow rate with little water pooling & puddling)

☐ Poor (does not allows water to drain, there is pooling & puddling)

14. Water source for cattle:

☐ Municipal ☐ Well ☐ Borehole ☐ River ☐ Dam

15. Segregation of cattle housing:

☐ separation between cows, heifers & calves

☐ absence of separation between cows, heifers and calves

16. Level of hygiene and sanitary conditions of cattle housing:

☐ Excellent (exceeds animal welfare minimum standards on housing)

☐ Good (satisfies animal welfare minimum standards on quality on housing)

☐ Poor (does not satisfy animal welfare minimum standards on housing)

17. Main reasons of selective culling of animals:

☐ Feet/claw disorders ☐ Udder disorders ☐ Metabolic and digestive disorders

☐ Fertility problems ☐ Others specify: ________________________________

18. Origin of replacement animals:

☐ Other farms ☐ Born on farm ☐ Both (other farms & born on farm)

If from other farms specify type of replacement animals:

☐ Pregnant cows/heifer ☐ Open cows/heifer ☐ Bulls

19. Any tests done to replacement animals before introduction?

☐ Yes ☐ No

If yes specify test: ____

20. Quarantine practiced when introducing new animals:

☐ Yes ☐ No

21. Any specific area or housing used for quarantine?

☐ Yes ☐ No

22. How long is quarantine?

☐ < 2 weeks ☐ 2 – 4 weeks ☐ > 4 weeks

23. Level of biosecurity of cattle husbandry practices on farm:

☐ High biosecurity (excellent control access, disinfectant footbaths, working fence)

☐ Moderate biosecurity (good control access, no disinfectant footbaths, occasionally working fences)

☐ Low biosecurity (no control access, no working fences)

24. Main health problems in the herd:

☐ Neonatal diarrhoea ☐ Bovine respiratory disease ☐ Metabolic diseases

☐ Parasitic diseases ☐ Mastitis ☐ Reproductive diseases ☐ Lameness

☐ Others

If others specify: ____

25. Implementation of disinfection, fumigation and rodent control measures:

☐ Yes ☐ No

26. Cattle kraaled/housed at night:

☐ Yes ☐ No

27. Presence of wildlife on farm:

☐ Yes ☐ No

If yes, specify: ____

☐ Wild canids ☐ Wild felids ☐ Wild herbivores

28. Presence of other domestic animals besides cattle on farm:

☐ Yes ☐ No

If yes: ☐ Dogs ☐ Cats ☐ Horses ☐ Poultry ☐ Small stock ☐ Pigs

☐ Others, specify ____

29. Number of dogs on farm: ☐ < 2 ☐ 2 – 5 ☐ > 5

30. Age of dogs:

☐ < 6 months ☐ 6 – 18 months ☐ > 18 months

31. New dogs bought on to farm in the past 24 months:

☐ Yes ☐ No

32. Dogs used as cattle working dogs:

☐ Yes ☐ No

33. Dogs have access to colostrum and or milk:

☐ Yes ☐ No

34. Dogs have access to uterine discharges or placental material or aborted foetuses:

☐ Yes ☐ No

35. Dogs defecate on or have access to pastures or feed troughs or feed storage areas:

☐ Yes ☐ No

36. Breeding service:

☐ Bull ☐ Artificial insemination

If bull any diagnosis of:

☐ Tritrichomoniasis ☐ Campylobacteriosis ☐ Bovine Viral Diarrhea

☐ Herpesvirus

37. Presence of calving location:

☐ Yes ☐ No

38. Use of calving location or pen to hospitalize sick animals

☐ Yes ☐ No

39. Management of colostrum:

☐ Pooled colostrum ☐ Calves suckle colostrum from dam

40. Milking type:

☐ Machine ☐ Hand

41. History of abortion on farm in the previous 24 months:

☐ Yes ☐ No

If yes what is the rate of abortion:

☐ < 2% ☐ 2% - 10% ☐ > 10%

42. Condition of aborted foetuses:

☐ Fresh ☐ Autolyzed ☐ Mummified

43. Birth of neurological or weak calves:

☐ Yes ☐ No

44. Annual rate of cows returning to oestrus post pregnancy:

Specify ___________________________________

45. Disposal of placenta or afterbirth or aborted foetuses done:

☐ Yes ☐ No

46. Destination of placenta or afterbirth or aborted foetus or dead animals:

☐ Destroyed on farm ☐ Destroyed off farm

☐ Sold or donated as animal feed/manufacturing of animal feed

47. ***Neospora caninum*** infection ever confirmed on farm?

☐ Yes ☐ No

if yes which diagnostic test and sample was used : __________________________________

48. Any other confirmed cause of reproductive failure confirmed on farm:

☐ Yes ☐ No

If yes (Specify) ____

49. Management of animal records and information:

☐ Software ☐ Use of log books ☐ No recording done

…………………………………… …..**END**………………………………………………

Thank you for your cooperation and time spent. Enjoy your day!

**Cattle**	**Identification** (Tag #)	**Sex (**Male / Female)	**Age group** (< 2 yrs; 2 – 4 yrs;< 4 yrs)	**Lactation** #	**Age of gestation** (1st,2rd,3rd)	**Parity** # (0;1; 2-4 ;<4)
1						
2						
3						
4						
5						
6						
7						
7						
8						
9						
10						
11						
12						
13						
14						
15						
16						
17						
18						
19						
20						
21						
22						
23						

24						
25						
26						
27						
28						
29						
30						
31						
32						
33						
34						
35						
