# Supplementary material for: Seroprevalence and associated risk factors for Neospora caninum infection in dairy cattle in South Africa
Source: Parasitol Res. 2024 Aug 14;123(8):298. doi: 10.1007/s00436-024-08309-8 (PMC11324681; doi:10.1007/s00436-024-08309-8)
Supplement: Supplementary file 2 — Supplementary file2 (PDF 141 KB) [file 436_2024_8309_MOESM2_ESM.pdf]

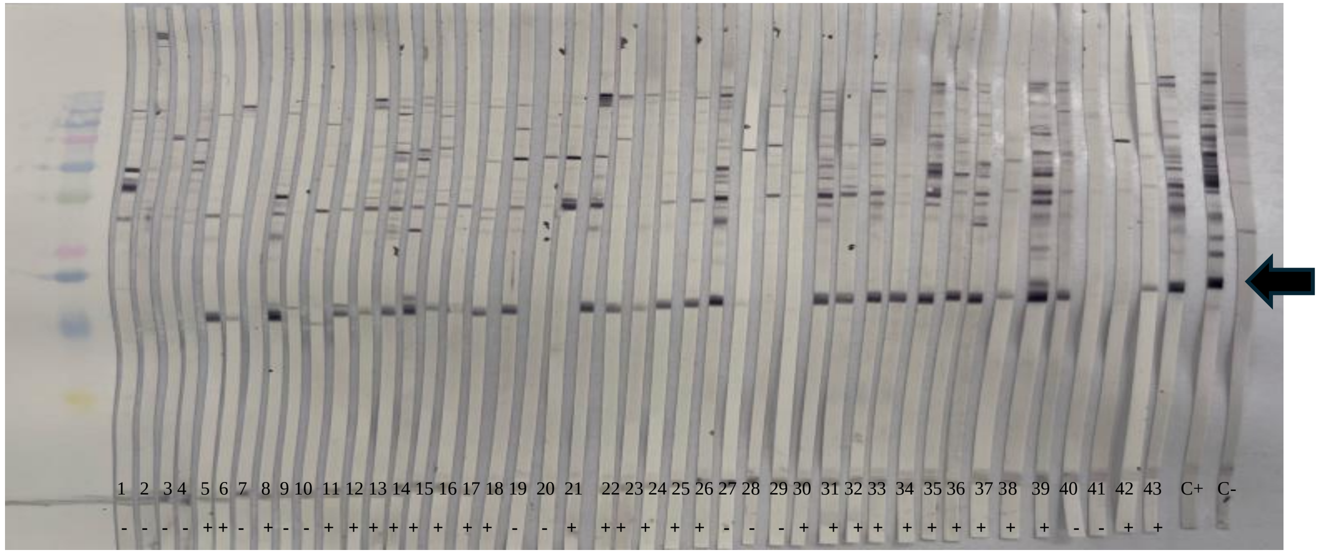

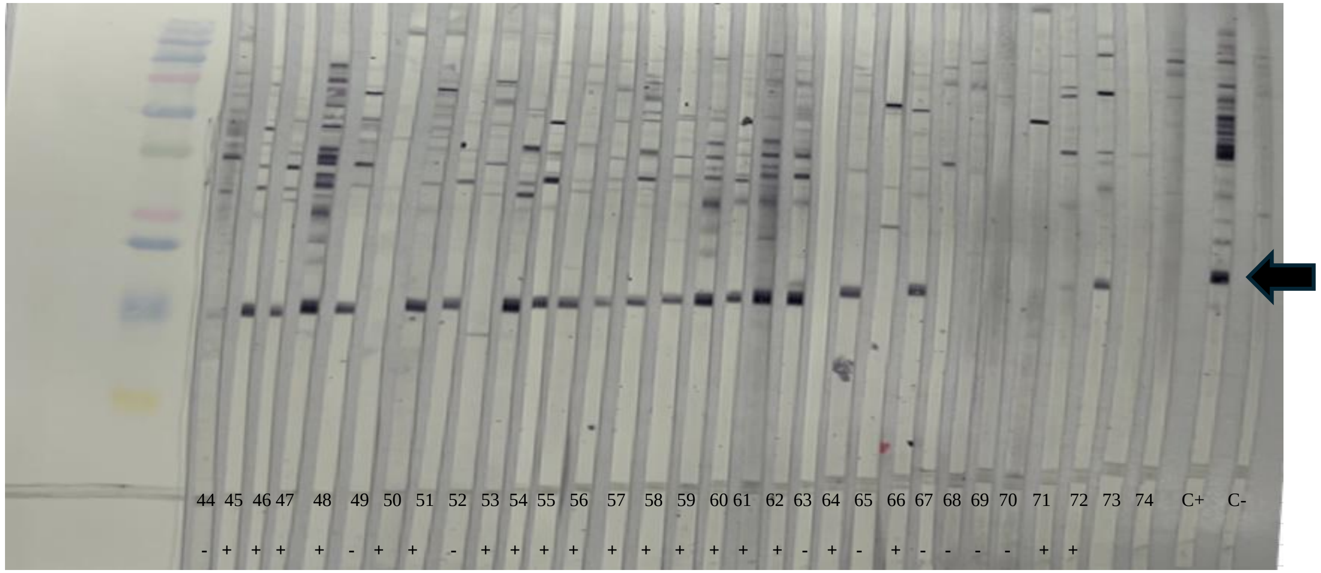

Western blot analysis of 74 dairy cattle serum samples using anti-IgG bovine secondary antibody. The recognition of 17-19 KDa antigenic fraction is indicated with an arrow. Positive and negative results are shown with “+” and “-” symbols, respectively, below lanes numbers.
